# Sensitive and Specific Peak Detection for SELDI-TOF Mass Spectrometry Using a Wavelet/Neural-Network Based Approach

**DOI:** 10.1371/journal.pone.0048103

**Published:** 2012-11-12

**Authors:** Vincent A. Emanuele II, Gitika Panicker, Brian M. Gurbaxani, Jin-Mann S. Lin, Elizabeth R. Unger

**Affiliations:** Chronic and Viral Diseases Branch, Division of High-Consequence Pathogens and Pathology, National Center for Emerging and Zoonotic Infectious Diseases, Centers for Disease Control and Prevention, Atlanta, Georgia, United States of America; Drexel University College of Medicine, United States of America

## Abstract

SELDI-TOF mass spectrometer's compact size and automated, high throughput design have been attractive to clinical researchers, and the platform has seen steady-use in biomarker studies. Despite new algorithms and preprocessing pipelines that have been developed to address reproducibility issues, visual inspection of the results of SELDI spectra preprocessing by the best algorithms still shows miscalled peaks and systematic sources of error. This suggests that there continues to be problems with SELDI preprocessing. In this work, we study the preprocessing of SELDI in detail and introduce improvements. While many algorithms, including the vendor supplied software, can identify peak clusters of specific mass (or m/z) in groups of spectra with high specificity and low false discover rate (FDR), the algorithms tend to underperform estimating the exact prevalence and intensity of peaks in those clusters. Thus group differences that at first appear very strong are shown, after careful and laborious hand inspection of the spectra, to be less than significant. Here we introduce a wavelet/neural network based algorithm which mimics what a team of expert, human users would call for peaks in each of several hundred spectra in a typical SELDI clinical study. The wavelet denoising part of the algorithm optimally smoothes the signal in each spectrum according to an improved suite of signal processing algorithms previously reported (the LibSELDI toolbox under development). The neural network part of the algorithm combines those results with the raw signal and a training dataset of expertly called peaks, to call peaks in a test set of spectra with approximately 95% accuracy. The new method was applied to data collected from a study of cervical mucus for the early detection of cervical cancer in HPV infected women. The method shows promise in addressing the ongoing SELDI reproducibility issues.

## Introduction

The data analysis pipeline following a SELDI study involves 1) preprocessing to produce quantified peak clusters, 2) manually validating peak clusters as a QC step, and 3) group analysis to find differences between cases and controls. The methodology for preprocessing SELDI involves multiple algorithmic steps, and has been reviewed in [1]. In particular, the goal of preprocessing is to detect peaks in individual spectra corresponding to proteins and to produce estimates of peak areas/concentrations while minimizing the effects of noise and artifacts. Validation and QC of the preprocessing steps is generally done manually and can be time-consuming. In addition, visual interpretation is not always objective and it is not uncommon for experts to have trouble reaching a consensus about the validity of a preprocessing result. However, this step is essential in order to reduce the chance that false positive and false negative peaks may bias the group comparison results. In a group analysis, peaks detected across multiple spectra are associated together to form peak clusters estimated to be from the same analyte (present/absent across samples, with varying peak area/concentration). Statistical techniques such as t-tests and Mann-Whitney U-tests are used to find peaks that are significantly different between groups. Out of these three major components in the SELDI clinical data analysis pipeline, the manual validation step can be especially laborious especially on heterogeneous clinical data that may contain subtypes. This ultimately limits the size of study feasible with SELDI.

In order to facilitate more accurate SELDI studies with larger sample sizes, we introduce a neural network model to improve the automation of the validation step along with major improvements to the LibSELDI preprocessing approach. The neural network is trained on approximately 4200 expert annotated peaks. In this way, the neural network mimics the validation behavior of our in-house scientists in a more automated and objective fashion. The algorithm improvements to LibSELDI include 1) a 650× speed up of the algorithm, 2) improved denoising to reduce artifacts, and 3) quantitation. These algorithm improvements are demonstrated on a pooled-sample dataset. Finally, the improved LibSELDI is combined with the neural network and tested on a pilot clinical dataset consisting of samples from two different stages of cervical neoplasia. We compare the results of the LibSELDI/neural network approach to the standard Ciphergen Express analytical software on both the QC samples and the clinical samples.

## Methods

### Ethics Statement

This research was approved by the Centers for Disease Control and Prevention's Institutional Review Board. Informed consent was obtained in writing from participants in the study.

Cervical mucous was collected from women enrolled as part of an ongoing study of cervical neoplasia [2]. Briefly, participants were non-pregnant, HIV-negative women, aged between 18–69 years, attending colposcopy clinics at urban public hospitals in Atlanta, Georgia, and Detroit, Michigan between December 2000 and June 2004. As previously described, at the time of colposcopy two Weck-Cel® sponges (Xomed Surgical Products, Jacksonville, FL) were placed, one at a time, into the opening of the cervical canal that leads to the cavity of the uterus (cervical os) to absorb cervical secretions [3]. The wicks were immediately placed on dry ice and stored at −80°C until processed. Preparation of the pooled QC sample has been previously described [3], [4]. Forty Weck-Cel® sponges with no visual blood contamination from 25 randomly selected subjects were extracted using M-PER^®^ buffer (Thermo Fisher Scientific, Rockford, IL) containing 0.15M NaCl and 1× protease inhibitor (Roche, Indianapolis, IN). The extracts were combined, aliquoted and stored at −80°C until assayed. Total protein content was measured using the Coomasie Plus™ kit (Thermo Fisher Scientific) as per the manufacturer's protocol. For the pilot clinical analysis we selected 16 non-dysplastic cervical mucosa controls (CIN0) and 8 cervical intraepithelial neoplasia grade III cases (CIN3) consisting of post-menopausal women matched for age and race, so as to minimize the confounding effects of varied stages of the menstrual cycle on protein profiles.

The Protein Biological System II-c™ mass spectrometer, with Protein Chip software (version 3.2) (Ciphergen Biosystems, Fremont, CA) was used to perform SELDI-TOF MS as described previously [5]. Protein chip surface preparation, sample application, wash, and application of matrix was automated using the Biomek® 2000 laboratory automation workstation (Beckman Coulter Inc., Fullerton, CA) as per manufacturer's instructions (Ciphergen). The All-in-one protein standard (Ciphergen) was run weekly on the NP-20 (normal phase) chip surface (Ciphergen) to be used for external mass calibration. The QC sample was included as one spot on at least one chip in each run. The prepared weak cation exchanger chips (CM10) evaluated were incubated with the sample for 1 h at room temperature (24°C±2) and washed three times at 5 min intervals with the CM10 low stringency binding buffer, followed by a final wash with ddH_2_O. In the case of NP-20 arrays, the surface was prepared with 3 μL ddH_2_O, and ddH_2_O was used for all washing steps. Chips were air-dried 30 min prior to the application of sinnapinic acid (SPA) matrix. The chips were analyzed on the SELDI-TOF instrument within 4 h of application of the matrix. The previously optimized instrument settings were used here [5]. Data collection was set to 150 kDa optimized for m/z between 3–30 kDa for the low mass range. The laser intensity was set at 185 with a detector sensitivity of 8 and number of shots averaged at 180 per spot for each sample. Two warming shots were fired at each position with the selected laser intensity +10. These were not included in the data collection. Data was exported to Ciphergen Express Client (CE, version 3.5) for further analysis. Data collection from start to finish took 2 weeks.

CE was used to preprocess the spectra following a modification of the standard operating procedure that has been developed in house and previously described [6]. Briefly, baseline correction, external calibration using protein standards, normalization using total ion current, and mass alignment were applied to all spectra. Peak detection was performed on this pre-processed data. Peaks from 3–30 kDa were detected by centroid mass, minimum percent threshold set to 10%, estimated peaks, and a mass window of 0.3%. Two different signal to noise settings were used for peak detection 1) First pass (S/N)  = 5, valley depth  = 3, Second pass (S/N)  = 3, valley depth  = 2; 2) First pass (S/N)  = 3, valley depth  = 2, no second pass. Group differences between Cin0 and Cin3 were estimated using the p-value wizard in CE. Significance of the median peak intensities between the 2 groups was calculated using the Mann-Whitney test as described in the Protein Chip Data Manager Software 3.5 Operation Manual.

Orthogonal wavelet transforms, while having excellent denoising properties in the mean-squared error sense, can sometimes produce artifacts. These artifacts appear in the data as localized ringing in the vicinity of high frequency components/discontinuities (the pseudo-Gibbs effect) and reconstruction errors containing imprints of the particular wavelet basis used with the transform. To address these issues, Coifman and Donoho introduced the concept of cycle-spinning [7]. Let denote the vector of raw intensities measured from a SELDI experiment, and let and be the circulant-shift operator and the wavelet-denoising operator, respectively. The “Cycle-Spinning” wavelet transform is defined by Coifman and Donoho as
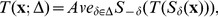
(1)where 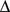 is a set of signal shifts. In other words, this framework is a shift-denoise-unshift-average approach [7]. Coifman and Donoho have shown that this approach suppresses the energy in artifacts. The cycle-spinning wavelet transform is also equivalent to the undecimated and translation-invariant wavelet transforms. Coombes et al. [8] have previously introduced the undecimated wavelet transform for application to SELDI data. Since this is a general framework and 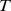 can represent any wavelet denoising operator, we extended the quadratic variance-based denoising of Emanuele and Gurbaxani [9] to use cycle spinning by applying (1) with 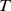 defined by eq. (10) of [9].

We designed and implemented a zero-phase, finite-impulse response (FIR) filter for LibSELDI (LS) to prepare processed spectra for quantification using peak heights or peak areas. While LS has been shown previously to perform well at resolving the mean m/z of peak clusters in a group of spectra, the denoised output of the modified Antoniadis-Sapatinas algorithm often decreases the peak heights. This effect was noted previously by Besbeas et al. [10]. The comparison paper by Cruz-Marcelo et al. [11] showed that different preprocessing techniques tend to be good at peak detection and peak quantification, respectively. This seems to imply that separate strategies are required for these preprocessing tasks. We designed the filter using the Parks-McLellan algorithm to give us good noise attenuation properties while maintaining the fidelity of the peak shape [12].

To automate peak validation, a feed-forward neural network with one hidden layer and sigmoid activation function was built in 4 steps: 1) a large set of manually validated peaks to use for model parameter estimation was created, 2) peaks were divided into training/validation/test sets according to a 50/25/25 percent split, 3) model parameters were estimated, 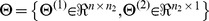, and regularization parameter, 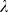, using the training and validation sets, and 4) the generalization/test error was estimated. A detailed review of neural networks can be found in [13]. In our model of peak validation, a peak had one of two states: 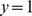 if validated and 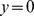 if discarded. The goal of the neural network was to take feature vectors 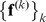 derived from local windows around a set of peak m/z predictions 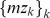 and produce a corresponding set of predictions 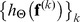 where 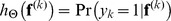. For training the network, let 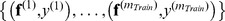 denote 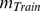 training examples. The regularized cross-entropy cost function corresponding to a neural network with known parameters and 1-hidden layer topology is
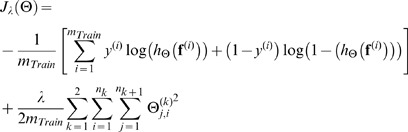
(2)where



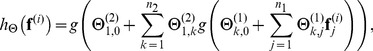
(3)

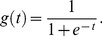
(4)


Note that in (2)–(4), 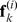 represents the 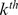 element of the 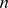 dimensional feature vector 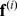. For a given 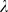, 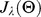 was minimized using the conjugate-gradient method (Polack-Ribiere) with step sizes selected by the slope-ratio method and Wolfe-Powell stopping criteria [14]. To fit the regularization parameter, we calculated the validation set classification error over a grid of 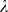 values and kept the 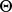 corresponding tos the 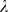 giving the lowest validation set classification error. With this best set of parameters, we evaluated the performance on the test set to estimate the expected performance on data that the neural network has not seen. Using the best 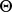, we validated a predicted peak cluster from a clinical experiment using the following procedure:

1. Let 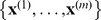 and 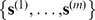 be a collection of raw and accompanying processed spectra, respectively, from LS. Define 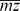 to be a mean peak cluster m/z value estimated to be present in the data using LS.
2. In a local window 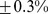 around 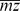, find if a local peak, 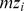 exists. If so, extract feature vector 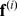 from 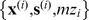, and add 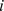 to set 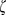. Repeat for all spectra 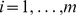.
3. For 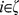, “look” at the peak with the neural network to validate the prediction. In other words, keep peak 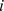 in the cluster if and only if 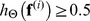.
4. Calculate the peak cluster prevalence as 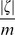, and extract peak height and peak area values for each peak that has been validated for use in the group analysis step later.

We used a dataset of spectra from 31 pooled cervical mucous QC samples to evaluate the ability of LS and CE to accurately find peak cluster mean m/z values corresponding to reproducible peaks. We define a reproducible peak as one that is present at the same m/z value (within 0.3% mass error tolerance) in 80% or more of the spectra. Two of the authors (VE, GP) visually inspected every reproducible peak predicted by each method adhering to the following protocol:

1. Size of window or zoom was ±2% of the m/z value of the peak.
2. Peaks were categorized separately as Confirmed or Rejected for the processed and raw spectra. Agreement was required between authors VE and GP for close calls.
3. If a peak was confirmed in the processed spectra but rejected in the raw spectra, the final consensus call was “reject”, as the peak could be an artifact introduced after processing.
4. Criteria for rejection were:
  - Peaks that were too broad at a given m/z.
  - Peaks that could not be distinguished from the noise of the surrounding regions.
  - A cluster is rejected if there were less than 24 spectra with good peaks (prevalence  = 24/30 = 80%).
  - Peak was clearly an artifact from the preprocessing step.

Once all reproducible peaks had a final annotation of confirmed or rejected, summary statistics were calculated to analyze the virtues of each approach. For each approach, we calculated.
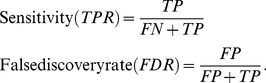
(5)


In this case, the estimated TP is the number of reproducible peaks predicted that were confirmed by visual inspection, the estimated FN is the number of confirmed peaks that were missed, and the estimated FP is the number of peaks that were rejected as false after visual inspection. In other words, sensitivity is the percentage of reproducible peaks that are confirmed via manual validation, while the false discovery rate is the percentage of reproducible peaks that were rejected after manual validation but predicted by CE or LS.

We tested both CE and LS on a pilot clinical data set containing 16 controls and 8 cases. In addition to estimating mean peak cluster m/z values accurately, clinical data presents the additional challenge of accurately estimating peak cluster prevalence and peak height/area measurements. Fisher-exact tests with mid-P correction were used to test for significant prevalence differences between cases and controls. T-tests and Mann-Whitney U tests were performed to find peak clusters with significant differences. Peak clusters with statistically significant behavior were qualitative reviewed to check for quality of the preprocessing and neural network-based validation results.

## Results

All samples were tested in duplicate and provided spectral profiles in the initial run. In the case of 3 samples, duplicate spectra were removed after initial analysis using CE as the normalization factor (NormF) was greater than average NormF+2σ generated with the batch analysis. These samples were rerun and replicate spectra used in further analysis. In the case of LS analysis, duplicate or replicate spectra were averaged to produce a single spectrum representing each subject prior to peak detection, unlike CE. In the CE analysis, duplicates or replicates peak heights were averaged after peak detection but before t-tests/group analysis.

**Figure 1:**
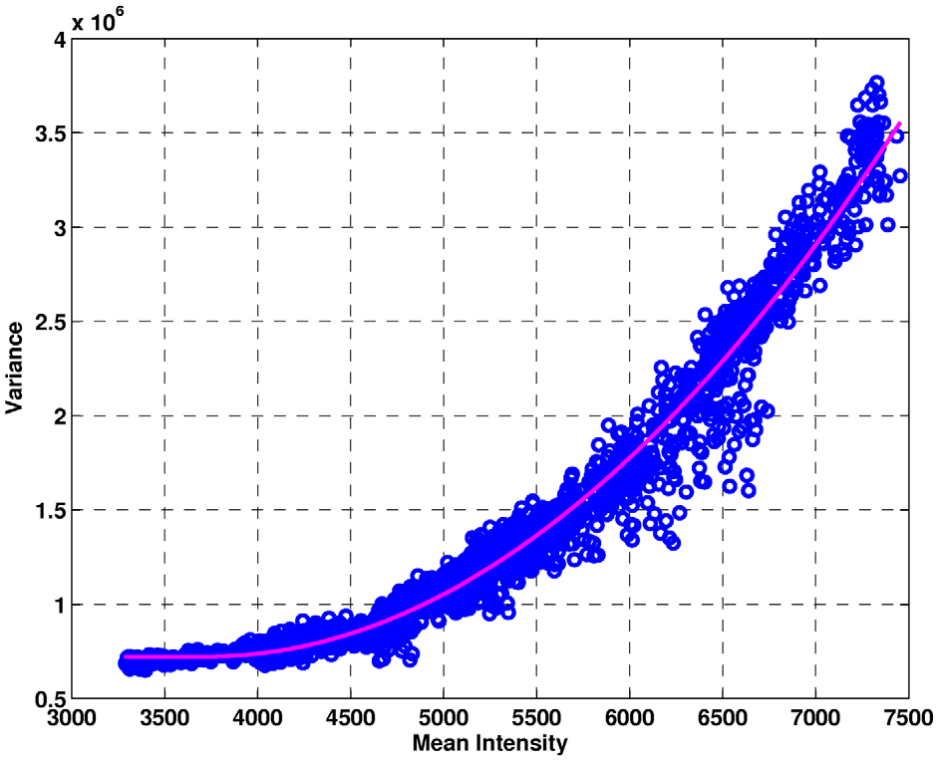
Quadratic detector response curve fit to data using space between the peaks of QC spectra.

We estimated the quadratic variance function (QVF) from spaces between the peaks of the QC data. Peak-free regions were selected by visual inspection, with the mean and variance at each point calculated across spectra. A quadratic detector response curve is fit to the mean/variance points using least squares, and results of the QVF estimation procedure are shown in Figure 1. The QVF is stored for use with LS as part of the modified Antoniadis-Sapatinas (mA-S) algorithm for denoising the QC and clinical spectra.

Extending the modified Antoniadis-Sapatinas algorithm to use cycle spinning required decreasing the computational complexity of the algorithm. For the implementation of the mA-S algorithm used in [9], [10], approximately 19.5 minutes of computing time using 10Gb of RAM was required to denoise a single spectrum. Coifman and Donoho showed that it is sufficient to compute 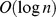 shifted transforms for a signal of length n. In a typical low-laser low-mass focused spectrum we have generated, 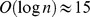, which means it would take almost 5 hours for a cycle-spinning mA-S algorithm to denoise a single spectrum. Five hours is enough time to manually process several spectra, so this result is unacceptable. We made the following observations about the computations:

1. The slow part of the computation was the default implementation for calculating the term 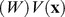 from Eq. (9) of [9].
2. Many computations were redundant in the 2-D wavelet decomposition used to calculate this term; i.e. the wavelet operator was the same for every spectrum and does not need to be recomputed every time.
3. The n × n wavelet operator was very sparse, containing approximately 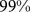 zeros.

Combining these facts we implemented a sparse matrix-based formulation of the problem to save memory and computation time rather than the standard 2-D filter bank decomposition. The wavelet transform matrix was constructed one time and a sparse-format matrix is stored off line. The sparse-matrix representation uses significantly less memory. Thus, in this implementation, every-time the 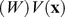 component needs to be computed, a sparse-matrix computed offline is read into memory and computation performed with a simple matrix multiply. This implementation resulted in a 640× speed up, as illustrated in Table 1. The time required to denoise a dataset of spectra from a group of 60 spectra decreased from ∼16 hours to less than 1.5 minutes.

**Table 1:** Processing time (in seconds) for denoising a single spectrum using different implementations of the modified Antoniadis-Sapatinas algorithm.

				n		
Implementation	2^10^	2^11^	2^12^	2^13^	2^14^	2^15^
Original	0.1235s	0.5066s	2.0476s	7.8817s	32.5274s	1155.85s
Gen-Sparse	0.0688s	0.2392s	0.9250s	9.1161s	23.3283s	97.5260s
Offline-Sparse	0.0089s	00174s	0.0407s	0.0999s	0.2177s	1.7856s

**Figure 2:**
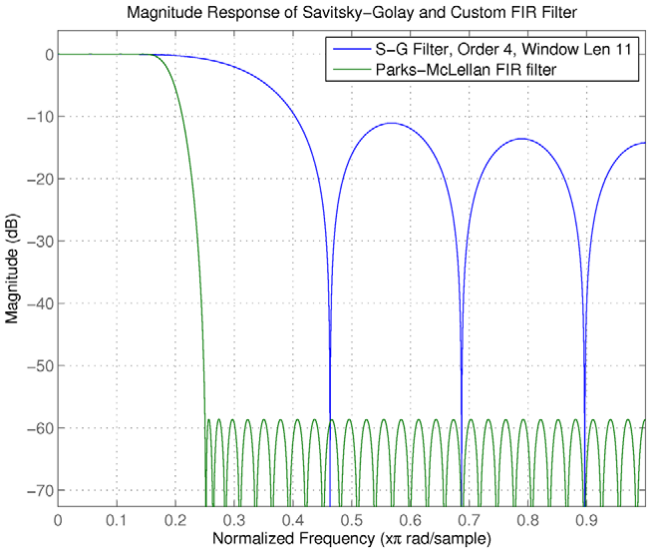
67^th^ order FIR filter frequency response designed for flat-pass band analogous to a Savitsky-Golay filter, but with better high-frequency noise suppression properties.

**Figure 3:**
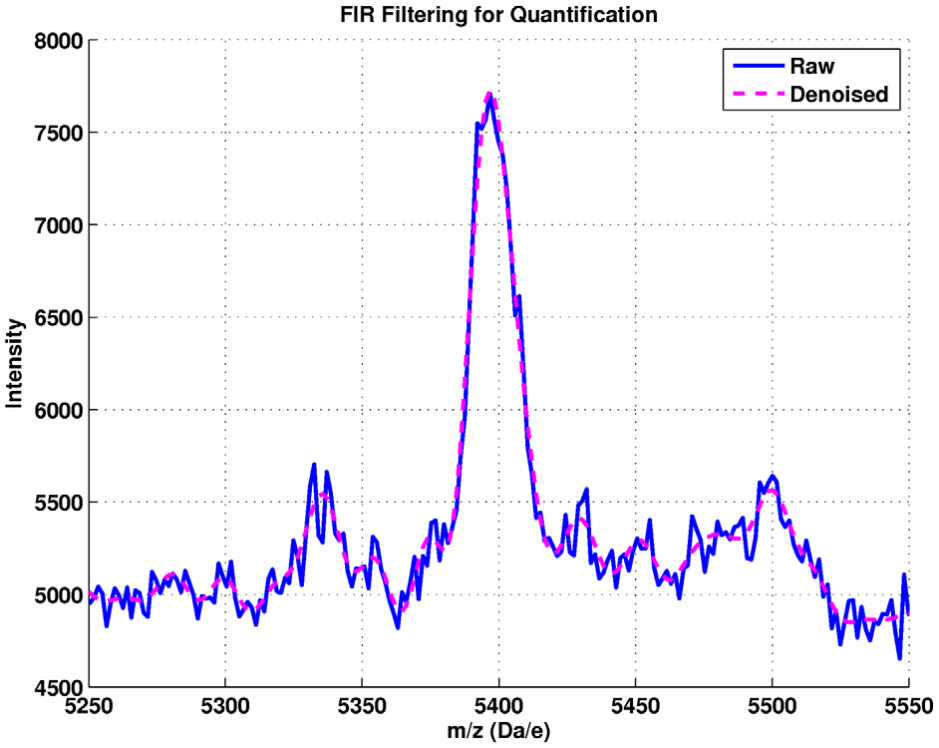
An example denoised peak using the FIR filter approach used for quantification. This is a typical example where the Antoniadis-Sapatinas denoising would find the peak but distort its peak height.

The FIR low-pass filter coefficients were estimated using the Parks-McClellan algorithm in MATLAB (firpm and firpmord functions). The specifications given the algorithm are: normalized frequency transition band between 0.15 and 0.25, pass-band ripple of 0.01, and stop band attenuation of 60 decibels (dB). The non-causal zero-phase implementation of the filter is used to prevent phase delays and preserve the locations of the peaks (filtfilt command in MATLAB)[15]. The order of the filter is 67. In Figures 2 and 3, we show the frequency response of the filter and smoothed output, respectively. Our FIR filter can be thought of as an extension of a Savitsky-Golay filter with greater noise suppression properties at high frequencies [16]. The smoothed spectra are much more visually consistent with that expected from visual observation by clinicians. The FIR smoothing procedure gives rise to ∼ 700 local maxima compared to the ∼ 150 given by the A-S algorithm, illustrating the tradeoff between peak detection performance and denoising performance between different smoothing approaches.

A feed-forward artificial neural network with one hidden layer was designed that validates peaks with (96.5%, 93.4%, 94.7%) accuracy on the training set, validation set, and test set, respectively. Peaks were detected in the QC data using LS with the most liberal settings possible so as to cast a wide net for all possible peak configurations and shapes in the data for the training process. We manually validated each prediction as a confirmed peak, a noisy/false prediction, or indeterminate. All indeterminate predictions were removed from the model estimation process. After removal of indeterminate cases, we had 4256 expert-annotated predictions, containing 57.07% percent “true” (confirmed peak) examples. We then randomly sampled the data into an approximate 50/25/25% split for the training set, validation set, and test set respectively. To construct the corresponding 62-dimensional feature vector 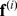 corresponding to a peak predicted at mass 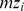, we considered the following intuition about how we manually QC peaks in-house.

1. For SELDI, all the information needed to make a judgment about peak validity is contained in a window 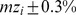.
2. When deciding whether a peak is acceptable or not, both the raw and processed spectra carry important information; a good peak shape in the processed spectra is not sufficient by itself if the corresponding raw spectrum is very noisy and of poor quality.

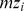
 may affect judgment due to changes in the theoretical peak shape as a function of mass.
4. Good peaks seem to look like a healthy concave quadratic centered at 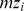, but not always.

These insights are applied to the following procedure used to construct the feature vector. Let 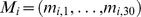 be a linear grid of 30 m/z points evenly spaced over the interval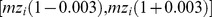.

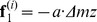
.(peak concavity measure, with parameter 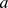 representing the quadratic coefficient of the best fit quadric curve for the raw intensities in the window centered at 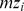).

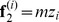
 (mass information, since peak shapes change with mass).

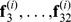
  =  the linearly interpolated intensities of the processed spectrum on support 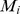.

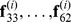
  =  the linearly interpolated intensities of the raw spectrum on support 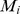.

We used a fully-connected feed-forward neural network with 63 input nodes, 21 hidden layer nodes, and 1 output layer node (including bias nodes). Thus, the parameter matrices had dimensions 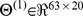,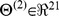. Before training, all features were standardized by subtracting the mean value in that dimension and dividing by the standard deviation (calculated across the training set). The normalization parameters are saved for use with the neural net on the validation data, test data, and clinical data. Regularization was used to control the complexity and avoid over-fitting. For each candidate value of 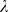 across a grid of points 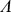 (between 0.003 and 10), the best-fit 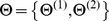 neural network parameters were found by using conjugate-gradient descent to minimize 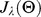 across the training set. The gradient of 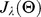 was calculated using the forward/back-propagation algorithm [13]. The optimization step was observed to converge after 400 iterations. For each 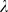, we calculated the classification error on the validation set, 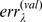. We selected our optimal neural network parameters as
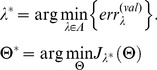
(6)where, in Eq. (6) it is understood that the estimate of 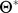 is most likely only a local minima. The trained neural network 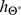 was evaluated on the independent test set and found to perform with a classification accuracy of 94.7%. By design, the test set was not used at any stage of the parameter-fitting process in order to ensure our test set classification accuracy estimate is an unbiased estimate for how the neural network will perform on peaks it has not “seen” at any stage of parameter fitting.

**Figure 4:**
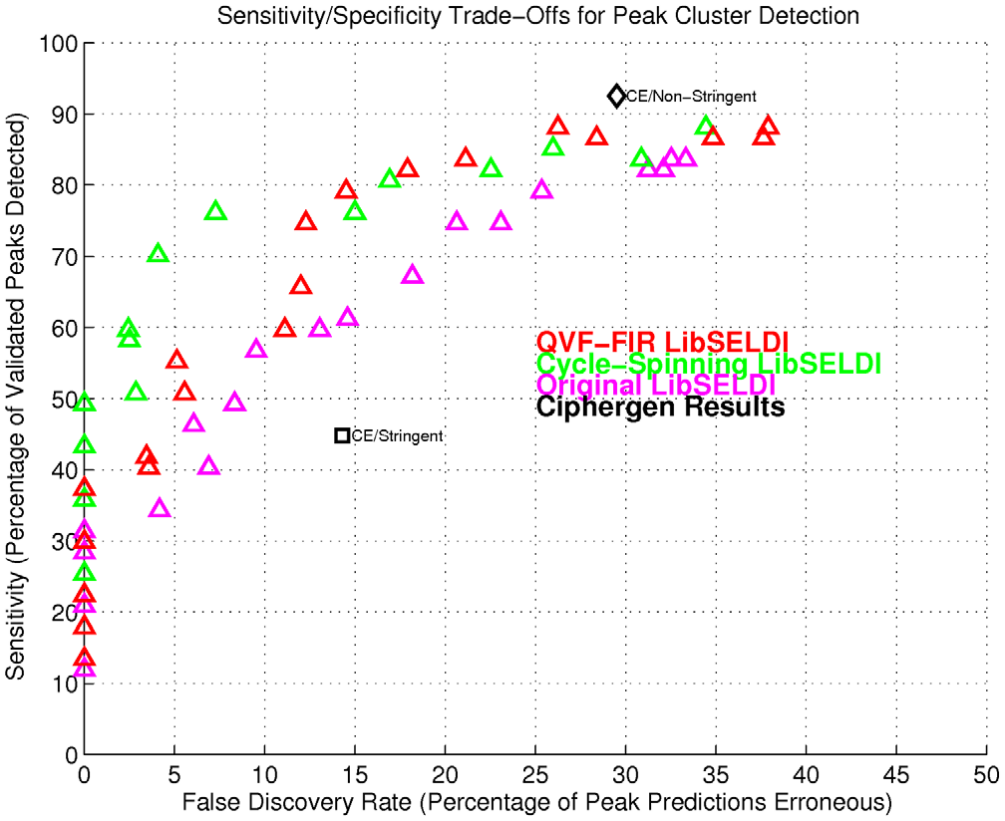
False-discovery rate and true-positive rate operating points showing various stages of improvement for LibSELDI.

LibSELDI showed improved sensitivity and specificity for detecting peak cluster mean m/z values corresponding to reproducible peaks in the pooled-sample QC data. Figure 4 shows the operating characteristics for LS at each iteration of improvement discussed in the Methods section. On QC spectra, LS recovered ∼50% percent of the true peak cluster m/z values without a mistake, and 70% at a 5% FDR. Operating points for Ciphergen Express using stringent (S/N 3/2) and non-stringent parameter settings (S/N 1) show a reference method for comparison. While peak cluster mean m/z values were reconstructed successfully, this benchmark did not show the accuracy of individual peak predictions within a cluster, a limitation of this approach. Resolving individual peak predictions within a cluster is critical for clinical group analysis and is discussed next.

**Figure 5:**
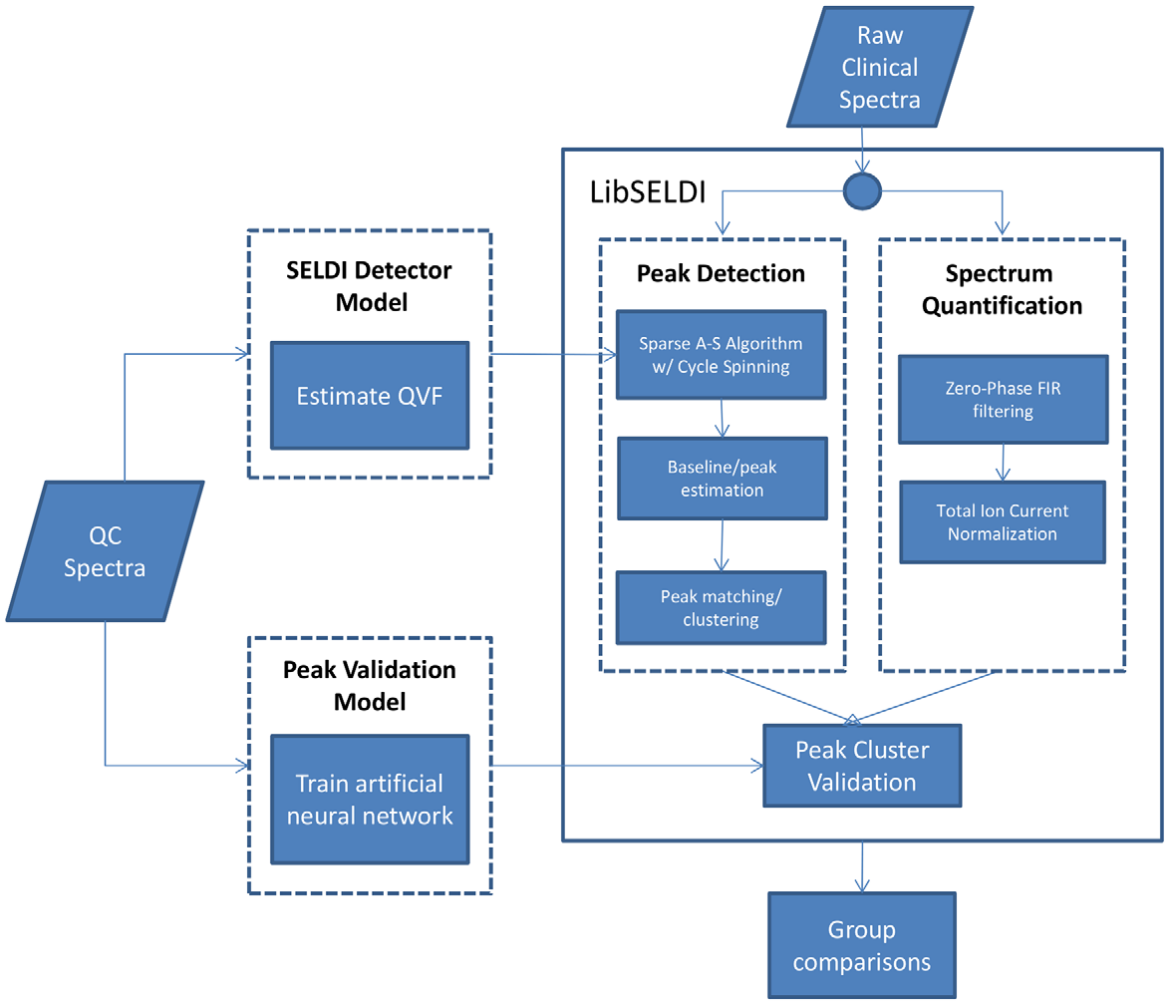
LibSELDI/neural network strategy for analyzing clinical spectra.

LS w/neural network validation found 124 peak clusters and resolved peak predictions accurately within clusters and CE with a SOP were applied to the pilot clinical mucous data. An overview of the LS/neural network strategy for analyzing clinical data is shown in Figure 5. Since the LS/neural network predictions were able to resolve more accurate peak predictions within clusters, this set the stage for a variety of analyses that would have been more difficult to carry out with Ciphergen Express alone. The results of t-tests, Mann-Whitney U-tests, and Fisher-exact tests with mid-P correction are shown in Tables 2 and 3. Only 2 p-values come in less than 0.01, and each test conducted tells a different story. There is some consistency in the tests with regards to which peak clusters tend to “look” different with respect to the various tests.

**Table 2:** CIN0 vs. CIN3 group tests (t-tests and Mann-Whitney U-tests) based on peak height and peak area measurements.

t-test, peak area			t-test, peak height			U-test, peak area			U-test, peak height		
* 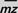 *	CIN0	CIN3	* 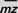 *	CIN0	CIN3	* 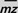 *	CIN0	CIN3	* 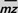 *	CIN0	CIN3
16054.5 Da	180 (8.6)	150 (1.1)	11821.9 Da	0.69 (0.12)	0.27 (0.07)	6912.8 Da	52.1 (0.6)	58.2 (3)	11821.9 Da	0.69 (0.1)	0.27 (0.1)
3017.1 Da	26.5 (1.6)	21.9 (0.6)	16054.5 Da	0.58 (0.09)	0.26 (0.003)	12680.8 Da	116.8 (3.8)	136.5 (8.0)	8287.7 Da	0.15 (0.03)	0.07 (0.008)
8287.7 Da	66.6 (0.9)	64.0 (0.4)	3017.1 Da	0.48 (0.12)	0.13 (0.03)	10427.1 Da	83.3 (1.9)	88.5 (2.8)	3682.3 Da	0.46 (0.06)	0.89 (0.16)
11821.9 Da	115.6 (5.1)	100.4 (3.8)	3682.3 Da	0.46 (0.06)	0.89 (0.16)	3682.3 Da	34.2 (1)	40.3 (2.3)	12680.8 Da	0.38 (0.07)	0.78 (0.15)
2887.8 Da	24.6 (0.5)	22.6 (0.6)	2887.8 Da	0.32 (0.04)	0.18 (0.03)	5647.0 Da	45.2 (0.6)	42.7 (0.8)	6912.8 Da	0.13 (0.01)	0.38 (0.13)
5849.7 Da	49.7 (2.2)	43.1 (1.0)	8287.7 Da	0.15 (0.03)	0.07 (0.01)						
3682.3 Da	34.2 (1.0)	40.3 (2.3)	5647.0 Da	0.22 (0.02)	0.14 (0.02)						
			12680.8 Da	0.38 (0.07)	0.78 (0.15)						

Showing quantification (SEM) for clusters with p-values less than 0.05.

**Table 3:** CIN0 vs. CIN3 prevalence differences scored using the Fisher-exact test with mid-P correction.

Fisher-exact tests with mid-P correction			
Cluster * 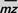 *	Prevalence, CIN3	Prevalence, CIN0	p-value
3017.1 Da	0.375	0.938	0.007

Showing only clusters with p-values less than 0.05.

**Figure 6:**
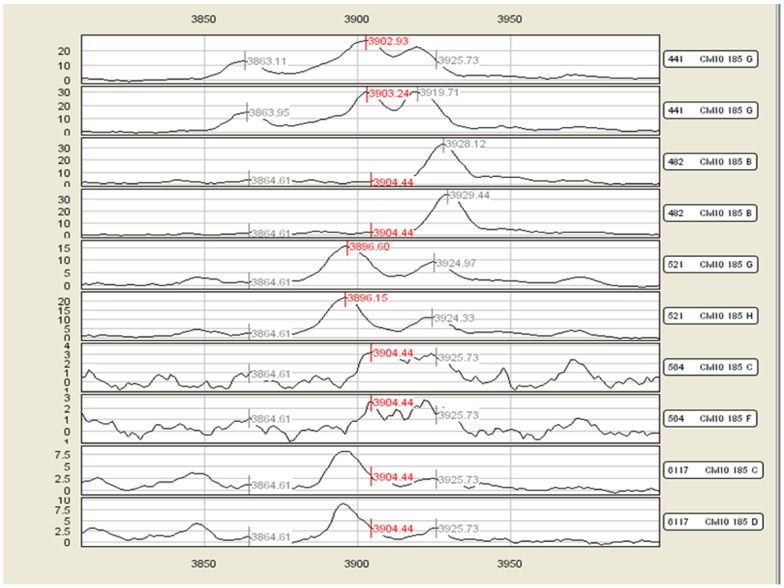
An example peak cluster output from Ciphergen Express v3.5.

With the different parameters used for peak detection, CE detected 106 (stringent) and 168 (non-stringent) peak clusters. Under the stringent parameter (S/N 5/3), 6 peak clusters were found to have differences (p-values less than 0.05, no multiple test correction) (Table 4) based on peak heights between CIN0 and CIN3 groups as opposed to 10 peak clusters with less stringent parameter (S/N 3/2). However, in several cases visual inspection of the spectra showed that peak detection was not accurate thereby leading to false readings of peak heights, as shown in Figure 6. This could be attributed to the need to include ‘estimated peaks’ as a peak detection parameter to enable calculation of p-values between sample groups in CE.

**Table 4:** CIN0 vs. CIN3 group tests (Mann-Whitney) based on peak height measurements under stringent condition (S/N 5/3) using Ciphergen Express.

Cluster * 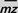 *	Average Peak Height (SD)	
	CIN3	CIN0
21663.4	0.843 (0.44)	0.429 (0.41)
3904.4	5.442 (7.75)	12.201(10.72)
7910.3	1.676 (1.36)	6.00 (5.63)
17205.5	0.121 (0.13)	0.570 (0.55)
8378.3	0.747 (0.35)	1.571 (1.26)
17341.8	0.171 (0.21)	0.57 (0.60)

Showing only clusters with p-values less than 0.05.

## Discussion

Neural networks were successful in their ability to automate the manual/visual validation step, mimicking the peak-calling performance of our in-house scientists with somewhere between 93%–95% accuracy. While this is very good classification performance for a complex task, we feel that for a true revolution to take place in SELDI preprocessing automation we would require a classifier with classification accuracies greater than 99.9%. After all, at our current accuracy rates, we still expect the neural-network validator to make 1–2 validation mistakes per cluster on our data. We feel strongly that if we could increase our training data by an order of magnitude (from ∼5000 peak examples to ∼50,000), the neural network approach we outlined could achieve such accuracy. With a classification accuracy of 99.9% we would only expect to make a single mistake validating a peak cluster representing a sample size of 1000! Such performance would enable the design of large studies with greater statistical power for making a biological discovery.

A case study in the challenges arising in biomarker discovery is the proteomics literature studying breast cancer. Starting in approximately 2002, breast cancer studies began to appear using the SELDI platform. Over the next several years, many studies followed using different specimens (mostly serum, plasma, or nipple aspirate fluid or NAF), on different groups of patients (early stage breast cancer, post-operative, benign breast cancer, those undergoing surgery, chemotherapy, radiation treatment, or some combination of the above), and some using the closely related MALDI instead of SELDI. Several proteins of interest began to emerge from the studies as being reproducible. Two helpful reviews by Calleson [17] and Gast [18] compiled some of the results. Specifically, three peaks of interest occurred in ≥5 studies that were subsequently identified via more specific protein chemistry methods: a neutrophil associated protein at ∼3440 Da, the inter-alpha-trypsin inhibitor heavy chain H4 (ITIH4) at ∼4300 Da, and the complement protein C3a des-arginine anaphylatoxin at ∼ 8940 Da. In all 3 cases, although multiple studies verified both the magnitude (reported as a p-value <0.05) and direction (over or under expressed in cancer) of the reported differences between groups, at least one confirmatory study using the same type of sample from similar groups of study subjects could not verify the magnitude of the difference, i.e. the p-value was no longer significant, or even the direction, i.e. the peak went from being significantly over expressed in cancer to significantly under expressed or vice versa [19]–[21]! The authors of these reviews and confirmatory studies therefore had to conclude in each case that more work was needed. Further preprocessing technique improvements enabling larger studies could help prevent some of the issues encountered by these studies.

Through a series of advancements to the different parts of the processing pipeline, LibSELDI has shown great promise for a level of detail in analysis of clinical data that was previously unavailable. The combination of the Antoniadis-Sapatinas algorithm-based denoising with an FIR filter designed for better noise suppression properties than popular Savitsky-Golay filter was a good combination of the strengths of each approach. The A–S algorithm has shown good performance for detecting and estimating peak cluster mean m/z values on simulated, pooled-sample QC, and clinical data. The tendency of the A–S denoising approach to unsatisfactorily alter the peak heights in the denoised spectra is balanced carefully with the FIR-filter based quantification step. We illustrated that the FIR-filtering step on its own would produce too many peak predictions, leading to many false positive peak clusters. By gluing these two methods together we have been able to capitalize on their respective strengths. We have confirmed that SELDI spectra are too inherently bumpy for a single denoising method to be superior at both the peak detection and quantification steps.

The computational tricks that enabled inclusion of the cycle-spinning variant of the modified A–S algorithm were also important, bringing LS a step closer towards enabling the use of SELDI for study designs with large sample size. We showed that a dataset that used to take 16 hours to process can now be processed in under 1.5 minutes. The addition of cycle-spinning reduced the energy in wavelet artifacts present in the denoised spectra, which led to increased sensitivity and specificity of the algorithm when benchmarked on the pooled-sample QC data.

The LibSELDI/neural-network validated peak clusters gave us higher quality predictions, allowing us to resolve individual peak predictions within clusters to a degree of accuracy that gave us fairly accurate measurements of the peak prevalence in each cluster and with respect to the case/control labels in the study. We were able to carry out a series of both parametric and non-parametric analyses based on peak height, peak area, and peak cluster prevalence. These analyses would have been impractical to conduct using the Ciphergen Express software alone. In all, 11 unique clusters came out of the analysis at a significance level below 0.05. Performing the study and analysis on a clinical set with larger sample size is a future work.

The current study contains several limitations that should be noted when interpreting the results. First, we have only shown results on a single sample medium analyzed on a single chip type. While we feel that LibSELDI algorithm extensions and neural-network validation model will extend to other Protein Chips and sample types (e.g. serum, plasma), we have not shown that in this paper. Also, the extension of the neural network to other chip and sample types may require adding significant additional training data to tune the neural network. In general, the baseline removal process has an effect on the quantification of peak heights and peak areas. To the authors' knowledge, there has been no study to date isolating the effect of baseline removal on peak quantitation. This holds for true for LS that we do not have a thorough understanding of the effect of the baseline removal technique used. Lastly, the sample size in our pilot clinical sample is too small to make any real biological conclusions. The small sample size was convenient for performing a preliminary evaluation of the LS and CE methodology on real clinical data. Follow up studies with larger samples sizes will be necessary in order to understand the performance limits of the methodology and to assess potential increases in statistical power for making biological discovery. Lastly, while we have qualitatively observed an improvement in the characterization of peak prevalence for peak clusters in clinical data, there is considerable room in the literature for a future work quantifying the performance of peak cluster composition/prevalence estimation.

The algorithmic and computational improvements in LibSELDI combined with the neural network-based peak cluster validation model moves us one step closer to larger SELDI experiments with greater chance of reproducible biological discovery.
